# Letter to the editor: Oropouche virus risk for European travellers to Cuba: an emerging public health concern

**DOI:** 10.2807/1560-7917.ES.2024.29.38.2400599

**Published:** 2024-09-19

**Authors:** Marta Giovanetti, Francesco Branda, Fabio Scarpa, Massimo Ciccozzi, Giancarlo Ceccarelli

**Affiliations:** 1 Sciences and Technologies for Sustainable Development and One Health, Università Campus Bio-Medico di Roma, Italy; 2 Instituto Rene Rachou, Fundação Oswaldo Cruz, Minas Gerais, Brazil; 3 Medical Statistics and Molecular Epidemiology, University Campus Bio-Medico, Rome, Italy; 4 Department of Biomedical Sciences, University of Sassari, Sassari, Italy; 5 Department of Public Health and Infectious Diseases, University of Rome Sapienza, Rome, Italy; 6 Azienda Ospedaliero Universitaria Umberto I, Rome, Italy; 7 Migrant and Global Health Research Organization (Mi-HeRO), Rome, Italy

**Keywords:** OROV, Emerging threat, global health

**To the Editor**: We read with great interest the report by Castelletti et al., documenting the first imported case of Oropouche virus (OROV) disease in Italy in travellers returning from Cuba [1]. On 27 May 2024, the Cuban Ministry of Public Health reported the first outbreaks of OROV disease in two provinces, Santiago de Cuba and Cienfuegos, with 74 confirmed cases [2]. Between June and July 2024, 19 imported cases of OROV disease were recorded in three European Union (EU) countries: Spain (12 cases), Italy (5 cases) and Germany (2 cases). Notably, 18 of these 19 cases were associated with travel to Cuba [3,4]. Given this information, the European Centre for Disease Prevention and Control (ECDC) assessed the risk of OROV infection for EU/European Economic Area (EU/EEA) citizens travelling to affected regions as moderate, considering the generally favourable prognosis for recovery [3].

However, in countries with larger OROV disease outbreaks, such as Brazil, where over 8,078 cases were confirmed by August 2024, wider viral circulation coupled with a lack of pre-existing immunity in newly affected populations may contribute to the observation of patients not only with a mild disease but also with more severe clinical outcomes [5]. This severity might be underestimated due to underdiagnosis and underreporting. Notably, fatal cases related to OROV infection have been described in young adults without comorbidities and in pregnant people, with evidence of vertical transmission and severe fetal complications (Figure).

**Figure:**
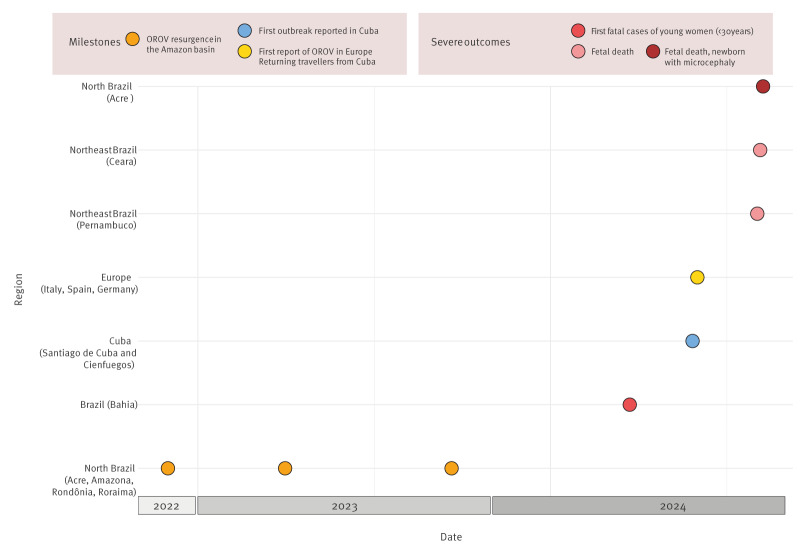
Timeline and geographic distribution of Oropouche virus (OROV) outbreaks and associated clinical outcomes, 2022–2024

Data for the figure were retrieved from [6-8].

Cuba is currently experiencing a surge in international tourist arrivals, influenced by the rise of cost-competitive vacation destinations in the region and increased air travel frequency. In 2023, Cuba recorded 2,436,979 non-resident international visitors, with a considerable portion originating from seven European countries: Spain, Germany, France, Italy, the United Kingdom (UK), Portugal and Poland, listed in descending order of volume. Preliminary data for 2024 suggest this is likely to continue [5].

Considering the current lack of comprehensive information, it is likely premature to definitively assess the risk for European travellers vacationing in Cuba. Initial epidemiological data indicate that tourist destinations are potentially at risk for OROV transmission. The actual number of OROV disease cases in travellers and tourists is likely underestimated due to mild symptomatology, self-limiting clinical course, potential underdiagnosis and limited diagnostic availability. However, the occurrence of severe OROV disease cases in countries experiencing larger outbreaks cannot be disregarded. Considering the volume of European tourism to Cuba, it is prudent to consider OROV in the differential diagnosis of severely ill patients returning from Cuba, particularly those without a diagnosis but presenting with compatible symptoms and in pregnant people returning from Cuba. This caution is advised until the epidemiological situation in Cuba is better defined and the objective risk of OROV infection is clarified.

## References

[1] Castilletti C Mori A Matucci A Ronzoni N Van Duffel L Rossini G Oropouche fever cases diagnosed in Italy in two epidemiologically non-related travellers from Cuba, late May to early June 2024. Euro Surveill. 2024;29(26):2400362. 10.2807/1560-7917.ES.2024.29.26.2400362 38940002 PMC11212459

[2] World Health Organization (WHO). Disease Outbreak News. Oropouche virus disease - Cuba. Geneva: WHO; 11 Jun 2024. Available at: https://www.who.int/emergencies/disease-outbreak-news/item/2024-DON521

[3] European Centre for Disease Prevention and Control (ECDC). Oropouche virus disease cases imported into the European Union. Stockholm: ECDC; 9 Aug 2024. Available from: https://www.ecdc.europa.eu/sites/default/files/documents/TAB-Oropouche-august-2024.pdf

[4] Iani FCM, Mota Pereira F, de Oliveira EC, Nascimento Rodrigues JT, Hoffmann Machado M, Fonseca V, et al. Rapid viral expansion beyond the Amazon Basin: increased epidemic activity of Oropouche virus across the Americas. 6 Aug 2024. medRxiv 10.1101/2024.08.02.24311415. 10.1101/2024.08.02.24311415

[5] Carmona Tamayo E, Perelló JL. Cuba en datos: El turismo internacional en Cuba cierra el 2023 con 2.4 millones de turistas. [Cuba in data: International tourism in Cuba closes 2023 with 2.4 million tourists]. Havana: Cubadebate; 2024. Spanish. Available from: http://www.cubadebate.cu/especiales/2024/01/19/cuba-en-datos-el-turismo-internacional-en-cuba-cierra-el-2023-con-24-millones-de-turistas/

[6] World Health Organization (WHO). Oropouche virus disease - Region of the Americas. Geneva: WHO; 23 Aug 2024. Available from: https://www.who.int/emergencies/disease-outbreak-news/item/2024-DON530

[7] Garcia Filho C, Lima Neto AS, Maia AMPC, Silva LOR, Cavalcante RdaC, Monteiro HdaS, et al. Vertical transmission of Oropouche virus in a newly affected extra-Amazon Region: a case study of fetal infection and death in Ceará, Brazil. 2 Sep 2024. SciELO Preprints 10.1590/SciELOPreprints.9667. 10.1590/SciELOPreprints.9667

[8] Bandeira AC, Barbosa ACFNdaS, Souza M, Saavedra RdaC, Pereira FM, Santos SPdeO, et al. Clinical profile of Oropouche fever in Bahia, Brazil: unexpected fatal cases. 16 Jul 2024. SciELO Preprints 10.1590/SciELOPreprints.9342. 10.1590/SciELOPreprints.9342

